# The genome sequence of the ichneumonid wasp, *Sussaba pulchella* (Holmgren, 1858) (Hymenoptera: Ichneumonidae)

**DOI:** 10.12688/wellcomeopenres.24968.1

**Published:** 2025-10-10

**Authors:** Ian Barnes, Chris Fletcher, Inez Januszczak, Gavin R. Broad, Liam M. Crowley, Natural History Museum Genome Acquisition Lab, Darwin Tree of Life Barcoding Collective, Wellcome Sanger Institute Tree of Life Management, Samples and Laboratory team, Wellcome Sanger Institute Scientific Operations: Sequencing Operations, Wellcome Sanger Institute Tree of Life Core Informatics team, Tree of Life Core Informatics collective, Darwin Tree of Life Consortium

**Affiliations:** 1 Natural History Museum, London, England, UK; 2 University of Oxford, Oxford, England, UK

**Keywords:** Sussaba pulchella, ichneumonid wasp, genome sequence, chromosomal, Hymenoptera

## Abstract

We present a genome assembly from an individual male *Sussaba pulchella* (ichneumonid wasp; Arthropoda; Insecta; Hymenoptera; Ichneumonidae). The genome sequence has a total length of 299.91 megabases. Most of the assembly (81.88%) is scaffolded into 15 chromosomal pseudomolecules. The mitochondrial genome has also been assembled, with a length of 34.69 kilobases. This assembly was generated as part of the Darwin Tree of Life project, which produces reference genomes for eukaryotic species found in Britain and Ireland.

## Species taxonomy

Eukaryota; Opisthokonta; Metazoa; Eumetazoa; Bilateria; Protostomia; Ecdysozoa; Panarthropoda; Arthropoda; Mandibulata; Pancrustacea; Hexapoda; Insecta; Dicondylia; Pterygota; Neoptera; Endopterygota; Hymenoptera; Apocrita; Ichneumonoidea; Ichneumonidae; Diplazontinae; *Sussaba*; *Sussaba pulchella* (Holmgren, 1858) (NCBI:txid662494)

## Background

*Sussaba pulchella* is a small (forewing ~4 mm) ichneumonid wasp of the subfamily Diplazontinae. As with all diplazontines for which host associations are known, *S. puchella* is a parasitoid of hoverfly (Diptera: Syrphidae) larvae. The biology of *S. pulchella* is poorly known. Although there are published host records from aphid-feeding hoverflies of the genus *Sphaerophoria*, such as *S. scripta* (Linnaeus) (e.g., Scott, 1939), these are probably due to misidentifications of other *Sussaba* species, particularly *S. flavipes*, which had previously been misidentified as *S. pulchella* (Diller, 1980). One species of *Sussaba* has been reared from larvae of *Pipizella* feeding on root-dwelling aphids ( Fitton & Rotheray, 1982), and the laterally compressed metasomas of *Sussaba* suggest that many of their hosts might be concealed in such a way, given that diplazontines which attack more exposed syrphid larvae tend to be frequently reared. Diplazontines emerge from the host puparium but oviposit in either the egg of the syrphid host (in the developing embryo), early or mid-instar larva. Host stage preference does not seem to be known for *Sussaba* but might be expected to be into the egg or early instar host, given the preferences of the most closely related genera with known host associations ( Klopfstein *et al.*, 2011).

Amongst Diplazontinae, *Sussaba* species can be recognised by the placement of the spiracle on the second metasomal segment, where it is on the laterotergite rather than the dorsal part of the tergite, as in all other Diplazontinae. The face of *Sussaba* species has two vertical grooves and is often unsculptured, thus resembling *Promethes*. Klopfstein (2014) provided keys to European Diplazontinae, including *Sussaba*, but identification of *Sussaba* species can be tricky. Males are more straightforward as the colour and shape of tyloids on the antennae tend to be species-specific. These tyloids are raised, ovoid areas of cuticle on the antennal flagellomeres and are involved in coiling the antennae around the female’s antennae, which is part of the courtship process in some clades of Diplazontinae ( Klopfstein *et al.*, 2010). Females can be separated from other species with the metasoma weakly laterally compressed by the colour pattern of the legs (coxae and trochanters all partly black), the distinct yellow ‘shoulder marks’ on the mesoscutum and the shining hind coxa. Males have uniformly dark brown tyloids, yellow and orange on the metasoma and fairly long antennae ( Klopfstein, 2014).

*Sussaba pulchella* is frequently collected and widespread across Britain, usually found by sweep netting. Its global range encompasses much of the Palaearctic, Nearctic and India ( Dasch, 1964; Klopfstein, 2014; Morley, 1913).

**Figure 1.**
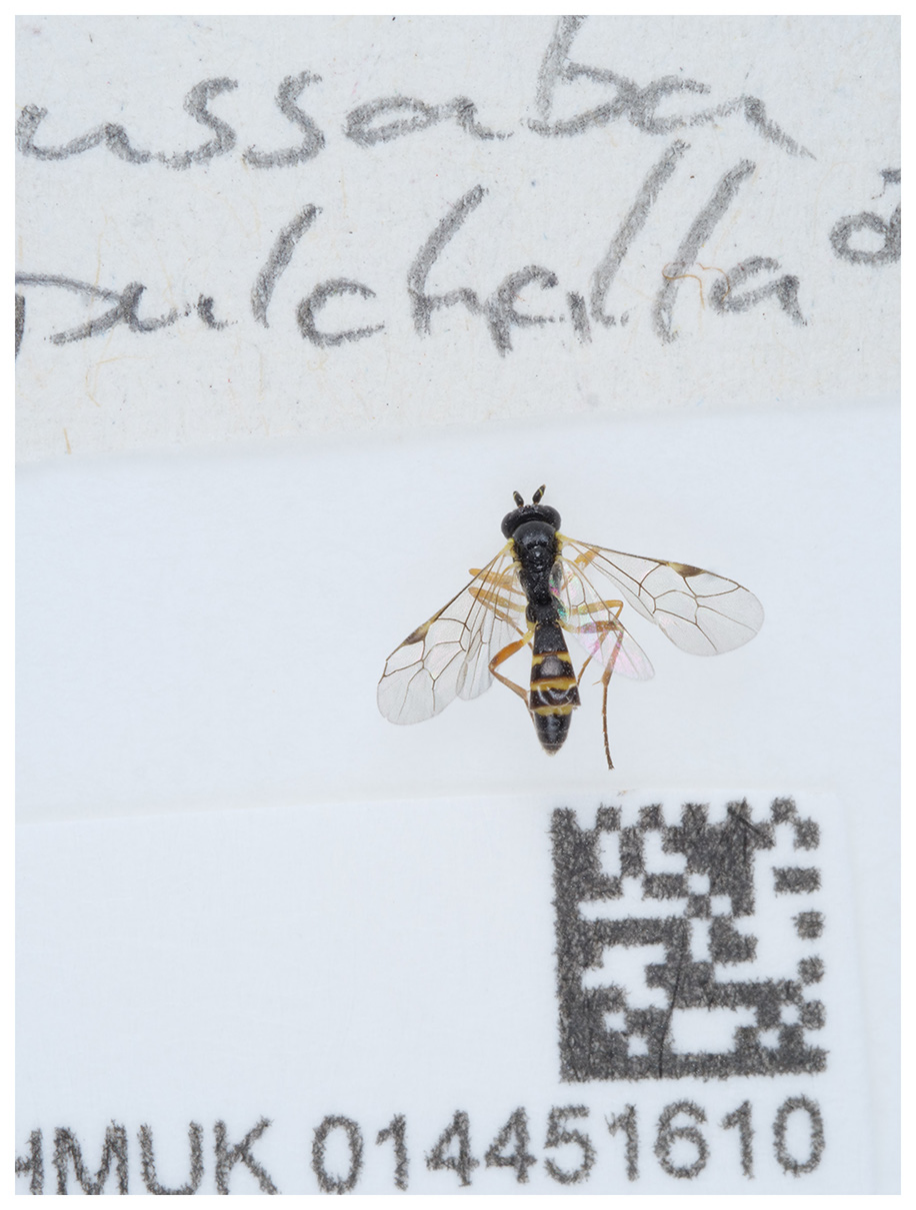
Photograph of the *Sussaba pulchella* (iySusPulc1) specimen used for genome sequencing.

We present a chromosome-level genome sequence for *Sussaba pulchella*, the first high-quality genome for the genus *Sussaba* (data obtained via NCBI datasets, O’Leary *et al.*, 2024). The assembly was produced using the Tree of Life pipeline from a specimen collected in Wytham Woods, England, UK ( Figure 1). This assembly was generated as part of the Darwin Tree of Life Project, which aims to generate high-quality reference genomes for all named eukaryotic species in Britain and Ireland to support research, conservation, and the sustainable use of biodiversity ( Blaxter *et al.*, 2022).

## Methods

### Sample acquisition and DNA barcoding

The specimen used for genome sequencing was an adult *Sussaba pulchella* (specimen ID NHMUK014451610, ToLID iySusPulc1; Figure 1), collected from Wytham Woods, Bert’s Pheasant Pen, England, United Kingdom (latitude 51.77, longitude –1.31) on 2021-09-02. The specimen was collected by Ian Barnes, Chris Fletcher, Inez Januszczak, Gavin Broad and Liam Crowley. For the Darwin Tree of Life sampling and metadata approach, refer to Lawniczak *et al*. (2022).

The initial identification was verified by an additional DNA barcoding process according to the framework developed by Twyford *et al.* (2024). A small sample was dissected from the specimen and stored in ethanol, while the remaining parts were shipped on dry ice to the Wellcome Sanger Institute (WSI) (see the protocol). The tissue was lysed, the COI marker region was amplified by PCR, and amplicons were sequenced and compared to the BOLD database, confirming the species identification ( Crowley *et al.*, 2023). Following whole genome sequence generation, the relevant DNA barcode region was also used alongside the initial barcoding data for sample tracking at the WSI ( Twyford *et al.*, 2024). The standard operating procedures for Darwin Tree of Life barcoding are available on protocols.io.

### Nucleic acid extraction

Protocols for high molecular weight (HMW) DNA extraction developed at the Wellcome Sanger Institute (WSI) Tree of Life Core Laboratory are available on protocols.io ( Howard *et al.*, 2025). The iySusPulc1 sample was weighed and triaged to determine the appropriate extraction protocol. Tissue from the whole organism was homogenised by powermashing using a PowerMasher II tissue disruptor. HMW DNA was extracted using the Automated MagAttract v2 protocol. We used centrifuge-mediated fragmentation to produce DNA fragments in the 8–10 kb range, following the Covaris g-TUBE protocol for ultra-low input (ULI). Sheared DNA was purified by automated SPRI (solid-phase reversible immobilisation). The concentration of the sheared and purified DNA was assessed using a Nanodrop spectrophotometer and Qubit Fluorometer using the Qubit dsDNA High Sensitivity Assay kit. Fragment size distribution was evaluated by running the sample on the FemtoPulse system.

### PacBio HiFi library preparation and sequencing

Library preparation and sequencing were performed at the WSI Scientific Operations core. Prior to library preparation, the DNA was fragmented to ~10 kb. Ultra-low-input (ULI) libraries were prepared using the PacBio SMRTbell® Express Template Prep Kit 2.0 and gDNA Sample Amplification Kit. Samples were normalised to 20 ng DNA. Single-strand overhang removal, DNA damage repair, and end-repair/A-tailing were performed according to the manufacturer’s instructions, followed by adapter ligation. A 0.85× pre-PCR clean-up was carried out with Promega ProNex beads.

The DNA was evenly divided into two aliquots for dual PCR (reactions A and B), both following the manufacturer’s protocol. A 0.85× post-PCR clean-up was performed with ProNex beads. DNA concentration was measured using a Qubit Fluorometer v4.0 (Thermo Fisher Scientific) with the Qubit HS Assay Kit, and fragment size was assessed on an Agilent Femto Pulse Automated Pulsed Field CE Instrument (Agilent Technologies) using the gDNA 55 kb BAC analysis kit. PCR reactions A and B were then pooled, ensuring a total mass of ≥500 ng in 47.4 μl.

The pooled sample underwent another round of DNA damage repair, end-repair/A-tailing, and hairpin adapter ligation. A 1× clean-up was performed with ProNex beads, followed by DNA quantification using the Qubit and fragment size analysis using the Agilent Femto Pulse. Size selection was performed on the Sage Sciences PippinHT system, with target fragment size determined by Femto Pulse analysis (typically 4–9 kb). Size-selected libraries were cleaned with 1.0× ProNex beads and normalised to 2 nM before sequencing.

The sample was sequenced using the Sequel IIe system (Pacific Biosciences, California, USA). The concentration of the library loaded onto the Sequel IIe was in the range 40–135 pM. The SMRT link software, a PacBio web-based end-to-end workflow manager, was used to set-up and monitor the run, and to perform primary and secondary analysis of the data upon completion.

### Hi-C

***Sample preparation and crosslinking***

The Hi-C sample was prepared from 20–50 mg of frozen whole organism tissue of the iySusPulc1 sample using the Arima-HiC v2 kit (Arima Genomics). Following the manufacturer’s instructions, tissue was fixed and DNA crosslinked using TC buffer to a final formaldehyde concentration of 2%. The tissue was homogenised using the Diagnocine Power Masher-II. Crosslinked DNA was digested with a restriction enzyme master mix, biotinylated, and ligated. Clean-up was performed with SPRISelect beads before library preparation. DNA concentration was measured with the Qubit Fluorometer (Thermo Fisher Scientific) and Qubit HS Assay Kit. The biotinylation percentage was estimated using the Arima-HiC v2 QC beads.

***Hi-C library preparation and sequencing***

Biotinylated DNA constructs were fragmented using a Covaris E220 sonicator and size selected to 400–600 bp using SPRISelect beads. DNA was enriched with Arima-HiC v2 kit Enrichment beads. End repair, A-tailing, and adapter ligation were carried out with the NEBNext Ultra II DNA Library Prep Kit (New England Biolabs), following a modified protocol where library preparation occurs while DNA remains bound to the Enrichment beads. Library amplification was performed using KAPA HiFi HotStart mix and a custom Unique Dual Index (UDI) barcode set (Integrated DNA Technologies). Depending on sample concentration and biotinylation percentage determined at the crosslinking stage, libraries were amplified with 10–16 PCR cycles. Post-PCR clean-up was performed with SPRISelect beads. Libraries were quantified using the AccuClear Ultra High Sensitivity dsDNA Standards Assay Kit (Biotium) and a FLUOstar Omega plate reader (BMG Labtech).

Prior to sequencing, libraries were normalised to 10 ng/μL. Normalised libraries were quantified again and equimolar and/or weighted 2.8 nM pools. Pool concentrations were checked using the Agilent 4200 TapeStation (Agilent) with High Sensitivity D500 reagents before sequencing. Sequencing was performed using paired-end 150 bp reads on the Illumina NovaSeq 6000.

### Genome assembly

Prior to assembly of the PacBio HiFi reads, a database of *k*-mer counts ( *k* = 31) was generated from the filtered reads using FastK. GenomeScope2 ( Ranallo-Benavidez *et al.*, 2020) was used to analyse the *k*-mer frequency distributions, providing estimates of genome size, heterozygosity, and repeat content.

The HiFi reads were assembled using Hifiasm ( Cheng *et al.*, 2021) with the --primary and --l0 options. The latter option is to switch off internal hifiasm purging for a haploid assembly. The Hi-C reads ( Rao *et al.*, 2014) were mapped to the primary contigs using bwa-mem2 ( Vasimuddin *et al.*, 2019), and the contigs were scaffolded in YaHS ( Zhou *et al.*, 2023) with the --break option for handling potential misassemblies. The scaffolded assemblies were evaluated using Gfastats ( Formenti *et al.*, 2022), BUSCO ( Manni *et al.*, 2021) and MERQURY.FK ( Rhie *et al.*, 2020).

The mitochondrial genome was assembled using MitoHiFi ( Uliano-Silva *et al.*, 2023), which runs MitoFinder ( Allio *et al.*, 2020) and uses these annotations to select the final mitochondrial contig and to ensure the general quality of the sequence.

### Assembly curation

The assembly was decontaminated using the Assembly Screen for Cobionts and Contaminants ( ASCC) pipeline. TreeVal was used to generate the flat files and maps for use in curation. Manual curation was conducted primarily in PretextView and HiGlass ( Kerpedjiev *et al.*, 2018). Scaffolds were visually inspected and corrected as described by Howe *et al*. (2021). Manual corrections included 58 breaks, 124 joins, and removal of four duplications. The curation process is documented at https://gitlab.com/wtsi-grit/rapid-curation. PretextSnapshot was used to generate a Hi-C contact map of the final assembly.

### Assembly quality assessment

The Merqury.FK tool ( Rhie *et al.*, 2020) was run in a Singularity container ( Kurtzer *et al.*, 2017) to evaluate *k*-mer completeness and assembly quality using the *k*-mer databases ( *k* = 31) computed prior to genome assembly. The analysis outputs included assembly QV scores and completeness statistics.

The genome was analysed using the BlobToolKit pipeline, a Nextflow implementation of the earlier Snakemake version ( Challis *et al.*, 2020). The pipeline aligns PacBio reads using minimap2 ( Li, 2018) and SAMtools ( Danecek *et al.*, 2021) to generate coverage tracks. It runs BUSCO ( Manni *et al.*, 2021) using lineages identified from the NCBI Taxonomy ( Schoch *et al.*, 2020). For the three domain-level lineages, BUSCO genes are aligned to the UniProt Reference Proteomes database ( Bateman *et al.*, 2023) using DIAMOND blastp ( Buchfink *et al.*, 2021). The genome is divided into chunks based on the density of BUSCO genes from the closest taxonomic lineage, and each chunk is aligned to the UniProt Reference Proteomes database with DIAMOND blastx. Sequences without hits are chunked using seqtk and aligned to the NT database with blastn ( Altschul *et al.*, 1990). The BlobToolKit suite consolidates all outputs into a blobdir for visualisation. The BlobToolKit pipeline was developed using nf-core tooling ( Ewels *et al.*, 2020) and MultiQC ( Ewels *et al.*, 2016), with containerisation through Docker ( Merkel, 2014) and Singularity ( Kurtzer *et al.*, 2017).

## Genome sequence report

### Sequence data

**Figure 2.**
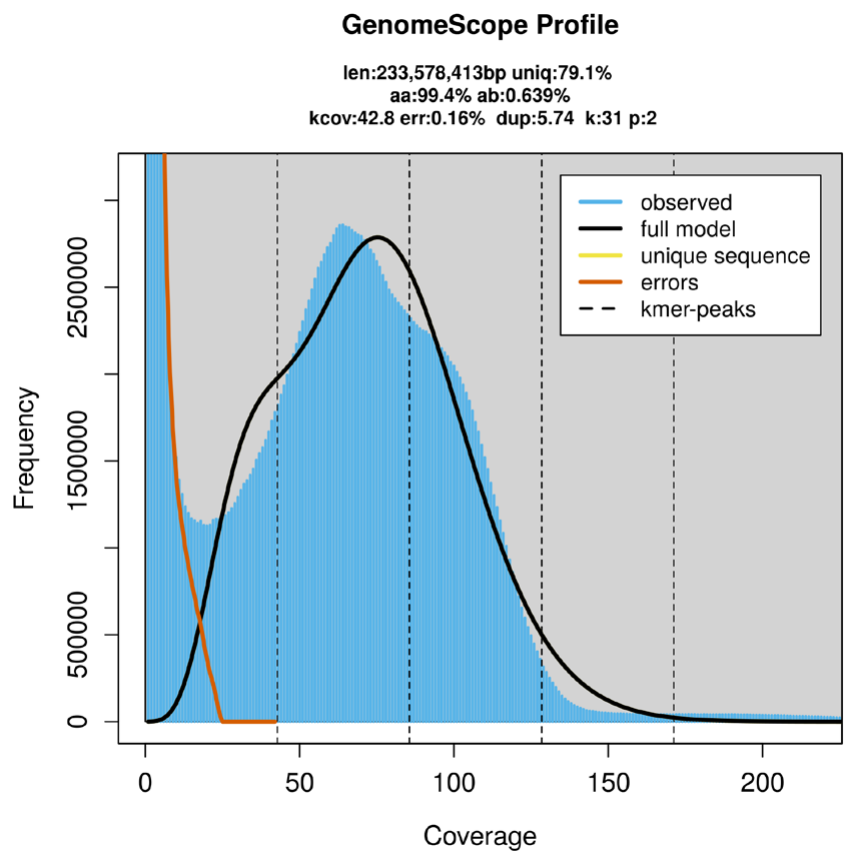
Frequency distribution of *k*-mers generated using GenomeScope2. The plot shows observed and modelled *k*-mer spectra, providing estimates of genome size, heterozygosity, and repeat content based on unassembled sequencing reads.

PacBio sequencing of the *Sussaba pulchella* specimen generated 22.63 Gb (gigabases) from 2.55 million reads, which were used to assemble the genome. GenomeScope2.0 analysis estimated the haploid genome size at 233.58 Mb, with a heterozygosity of 0.64% and repeat content of 21.52% ( Figure 2). These estimates guided expectations for the assembly. Based on the estimated genome size, the sequencing data provided approximately 86× coverage. Hi-C sequencing produced 70.97 Gb from 469.99 million reads, which were used to scaffold the assembly. Table 1 summarises the specimen and sequencing details.

**Table 1.** Specimen and sequencing data for BioProject PRJEB63414.

Platform	PacBio HiFi	Hi-C
**ToLID**	iySusPulc1	iySusPulc1
**Specimen ID**	NHMUK014451610	NHMUK014451610
**BioSample (source individual)**	SAMEA14448316	SAMEA14448316
**BioSample (tissue)**	SAMEA14448514	SAMEA14448514
**Tissue**	whole organism	whole organism
**Instrument**	Sequel IIe	Illumina NovaSeq 6000
**Run accessions**	ERR11593791	ERR11606303
**Read count total**	2.55 million	469.99 million
**Base count total**	22.63 Gb	70.97 Gb

### Assembly statistics

This assembly is from a haploid male specimen. The final assembly has a total length of 299.91 Mb in 1 379 scaffolds, with 1 639 gaps, and a scaffold N50 of 14.66 Mb ( Table 2).

**Table 2.** Genome assembly statistics.

**Assembly name**	iySusPulc1.1
**Assembly accession**	GCA_963971145.1
**Assembly level**	chromosome
**Span (Mb)**	299.91
**Number of chromosomes**	15
**Number of contigs**	3 018
**Contig N50**	0.2 Mb
**Number of scaffolds**	1 379
**Scaffold N50**	14.66 Mb
**Sex chromosomes**	N/A
**Organelles**	Mitochondrion: 34.69 kb

**Figure 3.**
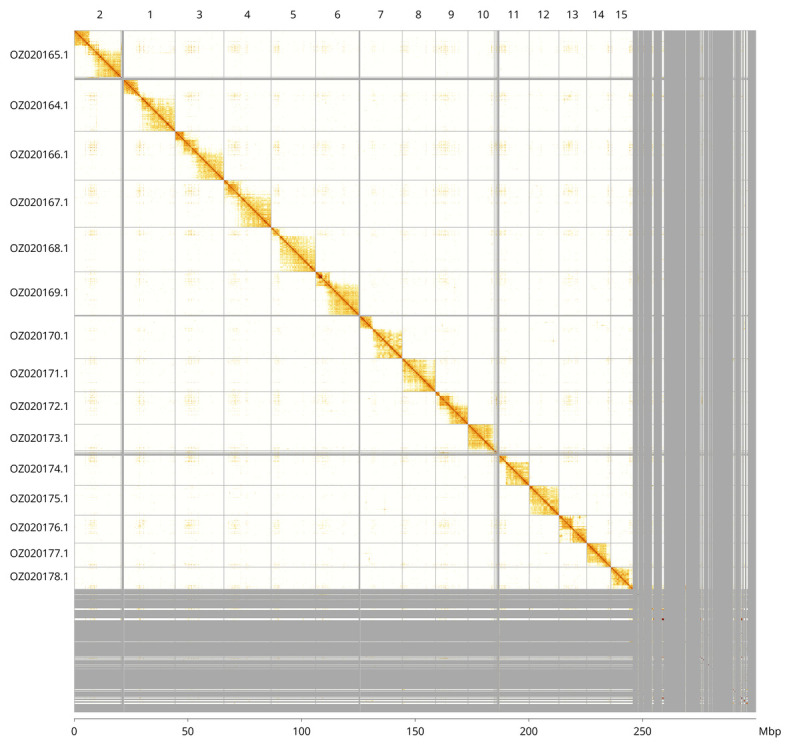
Hi-C contact map of the *Sussaba pulchella* genome assembly. Assembled chromosomes are shown in order of size and labelled along the axes, with a megabase scale shown below. The plot was generated using PretextSnapshot.

Most of the haploid assembly sequence (81.88%) was assigned to 15 chromosomal-level scaffolds. These chromosome-level scaffolds, confirmed by Hi-C data, are named according to size ( Figure 3; Table 3).

**Table 3.** Chromosomal pseudomolecules in the haploid genome assembly of *Sussaba pulchella* iySusPulc1.

INSDC accession	Molecule	Length (Mb)	GC%
OZ020164.1	1	21.77	39.50
OZ020165.1	2	22.63	39
OZ020166.1	3	21.43	40.50
OZ020167.1	4	20.76	40.50
OZ020168.1	5	19.49	40
OZ020169.1	6	19.49	40.50
OZ020170.1	7	18.54	39
OZ020171.1	8	14.66	39.50
OZ020172.1	9	14.22	38.50
OZ020173.1	10	13.74	39.50
OZ020174.1	11	13.18	40
OZ020175.1	12	13.08	39
OZ020176.1	13	12.23	40
OZ020177.1	14	10.62	38.50
OZ020178.1	15	9.71	38

The mitochondrial genome was also assembled. This sequence is included as a contig in the multifasta file of the genome submission and as a standalone record.

**Figure 4.**
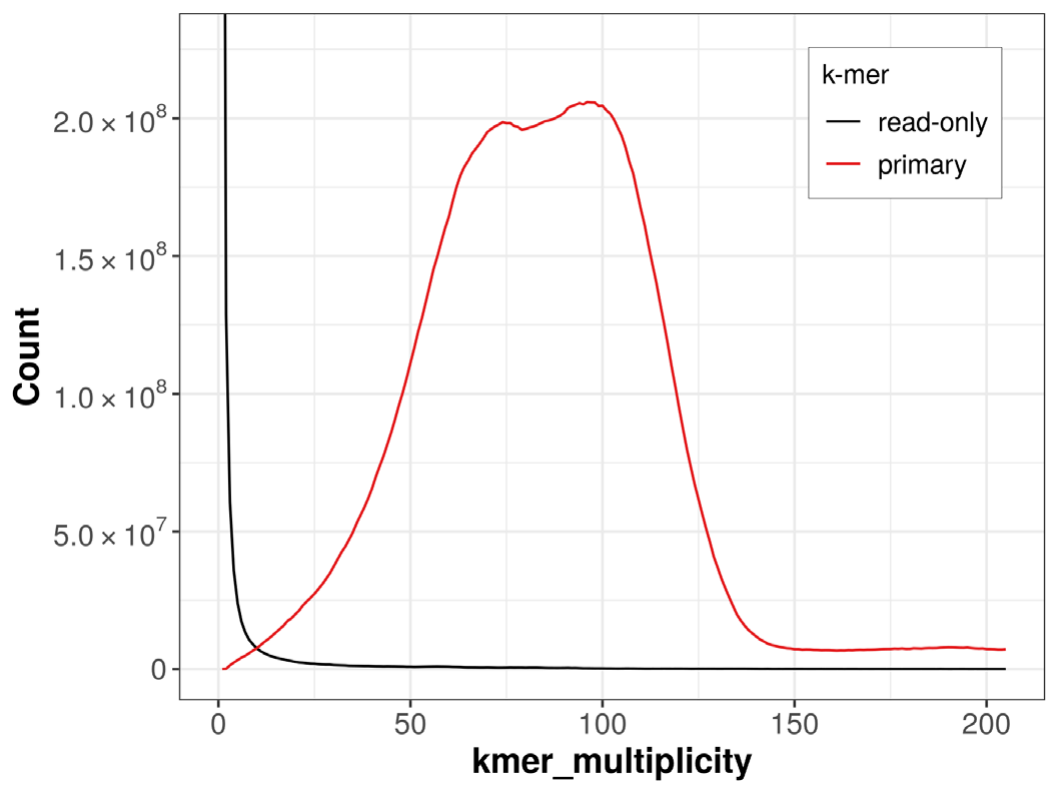
Evaluation of *k*-mer completeness using MerquryFK. This plot illustrates the recovery of *k*-mers from the original read data in the final assembly. The horizontal axis represents *k*-mer multiplicity, and the vertical axis shows the number of *k*-mers. The black curve represents *k*-mers that appear in the reads but are not assembled. The red curve corresponds to *k*-mers present in the haploid assembly.

The haploid assembly achieves an estimated QV of 55.3. The *k*-mer completeness is 98.89% for the haploid assembly ( Figure 4).

**Figure 5.**
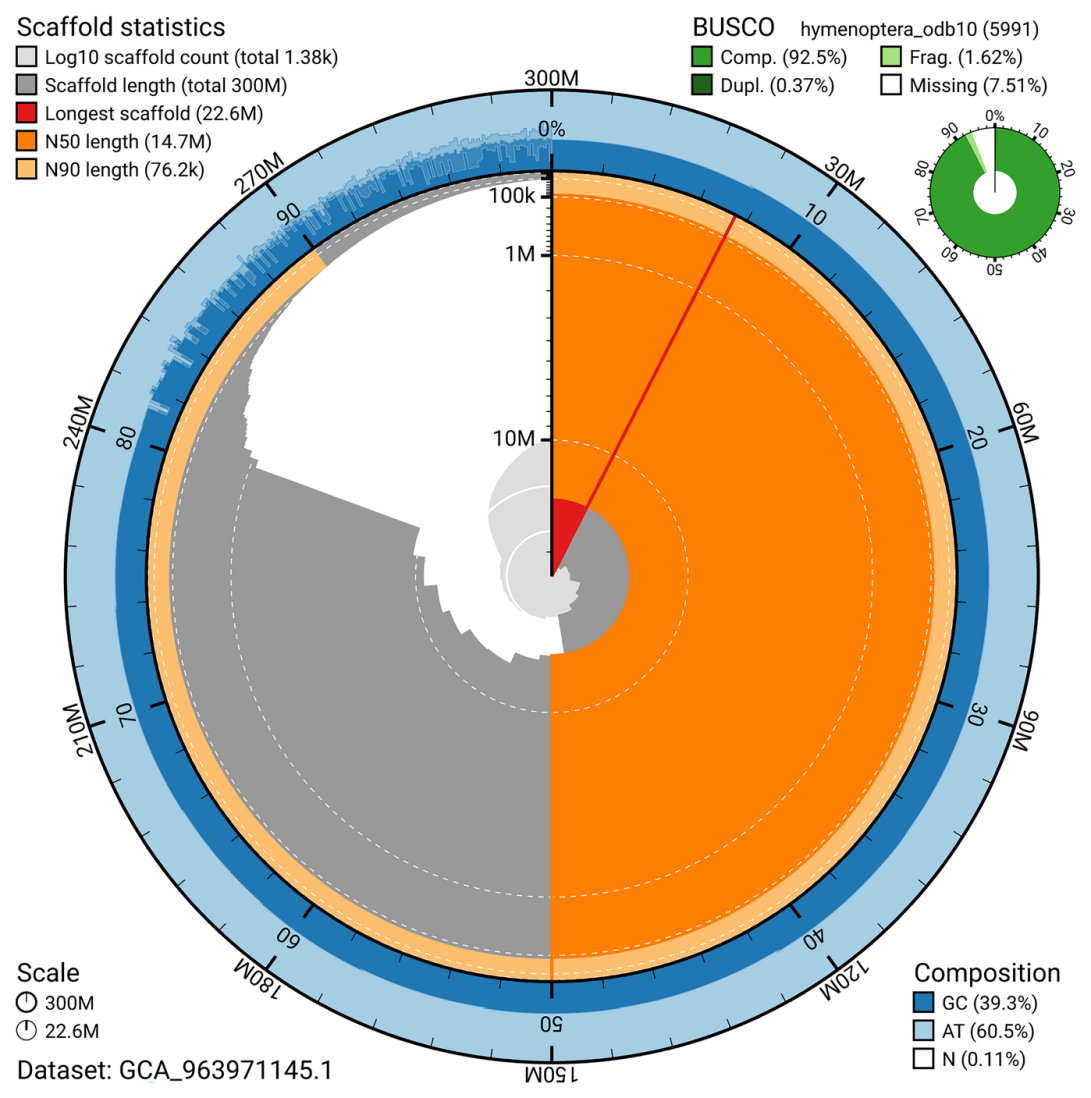
Assembly metrics for iySusPulc1.1. The BlobToolKit snail plot provides an overview of assembly metrics and BUSCO gene completeness. The circumference represents the length of the whole genome sequence, and the main plot is divided into 1 000 bins around the circumference. The outermost blue tracks display the distribution of GC, AT, and N percentages across the bins. Scaffolds are arranged clockwise from longest to shortest and are depicted in dark grey. The longest scaffold is indicated by the red arc, and the deeper orange and pale orange arcs represent the N50 and N90 lengths. A light grey spiral at the centre shows the cumulative scaffold count on a logarithmic scale. A summary of complete, fragmented, duplicated, and missing BUSCO genes in the hymenoptera_odb10 set is presented at the top right. An interactive version of this figure can be accessed on the BlobToolKit viewer.

**Figure 6.**
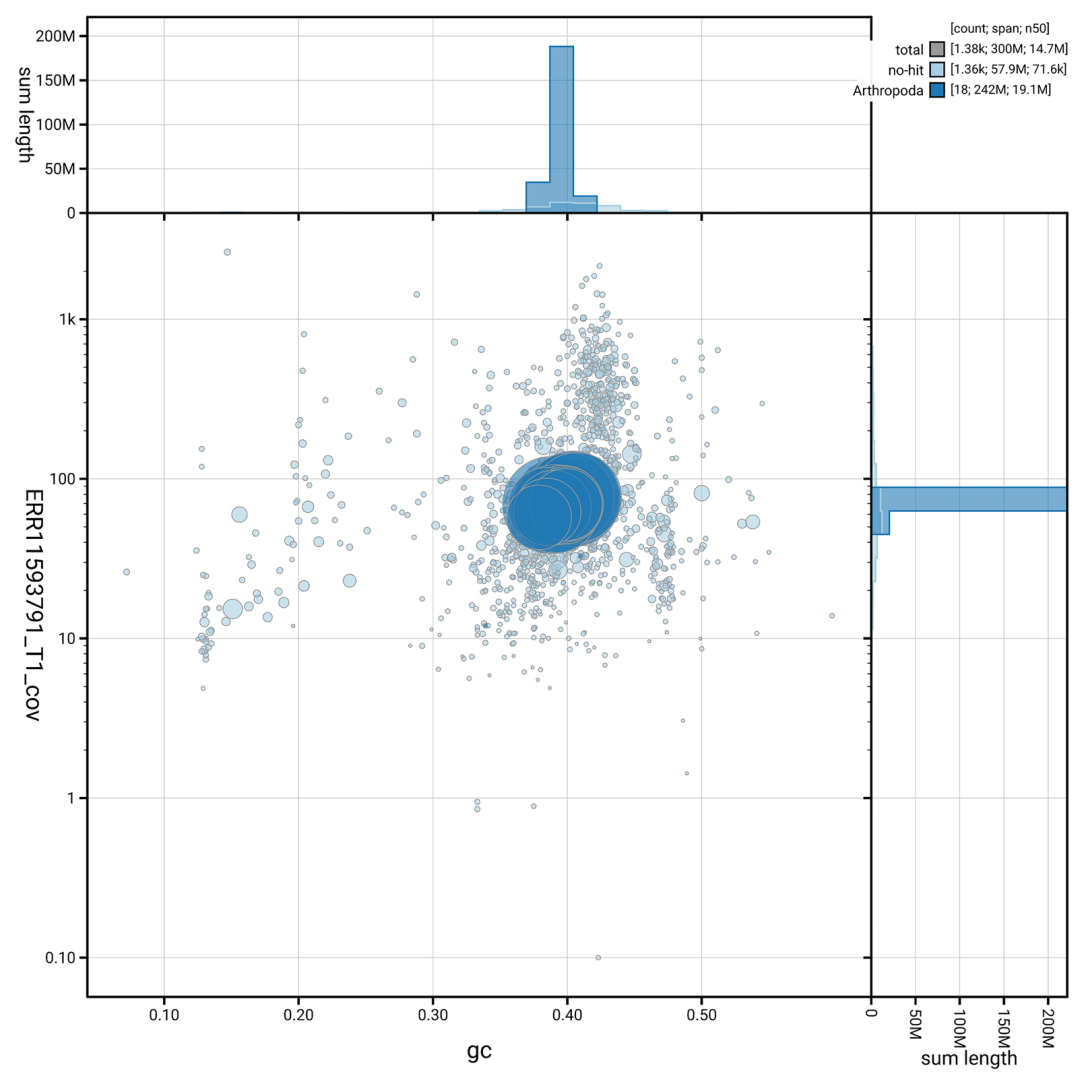
BlobToolKit GC-coverage plot for iySusPulc1.1. Blob plot showing sequence coverage (vertical axis) and GC content (horizontal axis). The circles represent scaffolds, with the size proportional to scaffold length and the colour representing phylum membership. The histograms along the axes display the total length of sequences distributed across different levels of coverage and GC content. An interactive version of this figure is available on the BlobToolKit viewer.

BUSCO v.5.5.0 analysis using the hymenoptera_odb10 reference set ( *n* = 5 991) identified 92.5% of the expected gene set (single = 92.1%, duplicated = 0.4%). The snail plot in Figure 5 summarises the scaffold length distribution and other assembly statistics for the primary assembly. The blob plot in Figure 6 shows the distribution of scaffolds by GC proportion and coverage.

Table 4 lists the assembly metric benchmarks adapted from Rhie *et al.* (2021) the Earth BioGenome Project Report on Assembly Standards September 2024. The EBP metric, calculated for the haploid assembly, is **5.7.Q55**.

**Table 4.** Earth Biogenome Project summary metrics for the *Sussaba pulchella* assembly.

Measure	Value	Benchmark
EBP summary (primary)	5.7.Q55	6.C.Q40
Contig N50 length	0.20 Mb	≥ 1 Mb
Scaffold N50 length	14.66 Mb	= chromosome N50
Consensus quality (QV)	55.3	≥ 40
*k*-mer completeness	98.89%	95%
BUSCO	C:92.5% [S:92.1%; D:0.4%]; F:1.6%; M:5.9%; n:5 991	S > 90%; D < 5%
Percentage of assembly assigned to chromosomes	81.88%	≥ 90%

### Wellcome Sanger Institute – Legal and Governance

The materials that have contributed to this genome note have been supplied by a Darwin Tree of Life Partner. The submission of materials by a Darwin Tree of Life Partner is subject to the **‘Darwin Tree of Life Project Sampling Code of Practice’**, which can be found in full on the Darwin Tree of Life website. By agreeing with and signing up to the Sampling Code of Practice, the Darwin Tree of Life Partner agrees they will meet the legal and ethical requirements and standards set out within this document in respect of all samples acquired for, and supplied to, the Darwin Tree of Life Project. Further, the Wellcome Sanger Institute employs a process whereby due diligence is carried out proportionate to the nature of the materials themselves, and the circumstances under which they have been/are to be collected and provided for use. The purpose of this is to address and mitigate any potential legal and/or ethical implications of receipt and use of the materials as part of the research project, and to ensure that in doing so we align with best practice wherever possible. The overarching areas of consideration are:

•   Ethical review of provenance and sourcing of the material

•   Legality of collection, transfer and use (national and international)

Each transfer of samples is further undertaken according to a Research Collaboration Agreement or Material Transfer Agreement entered into by the Darwin Tree of Life Partner, Genome Research Limited (operating as the Wellcome Sanger Institute), and in some circumstances, other Darwin Tree of Life collaborators.

## Data Availability

European Nucleotide Archive: Sussaba pulchella. Accession number PRJEB63414. The genome sequence is released openly for reuse. The *Sussaba pulchella* genome sequencing initiative is part of the Darwin Tree of Life Project (PRJEB40665) and the Sanger Institute Tree of Life Programme (PRJEB43745). All raw sequence data and the assembly have been deposited in INSDC databases. The genome will be annotated using available RNA-Seq data and presented through the Ensembl pipeline at the European Bioinformatics Institute. Raw data and assembly accession identifiers are reported in Table 1 and Table 2. Production code used in genome assembly at the WSI Tree of Life is available at https://github.com/sanger-tol. Table 5 lists software versions used in this study.

## Author information

Contributors are listed at the following links:

•   Members of the Natural History Museum Genome Acquisition Lab

•   Members of the Darwin Tree of Life Barcoding collective

•   Members of the Wellcome Sanger Institute Tree of Life Management, Samples and Laboratory team

•   Members of Wellcome Sanger Institute Scientific Operations – Sequencing Operations

•   Members of the Wellcome Sanger Institute Tree of Life Core Informatics team

•   Members of the Tree of Life Core Informatics collective

•   Members of the Darwin Tree of Life Consortium

## References

[ref-1] Allio R Schomaker-Bastos A Romiguier J : MitoFinder: efficient automated large-scale extraction of mitogenomic data in target enrichment phylogenomics. *Mol Ecol Resour.* 2020;20(4):892–905. 10.1111/1755-0998.13160 32243090 PMC7497042

[ref-2] Altschul SF Gish W Miller W : Basic Local Alignment Search Tool. *J Mol Biol.* 1990;215(3):403–410. 10.1016/S0022-2836(05)80360-2 2231712

[ref-3] Bateman A Martin MJ Orchard S : UniProt: the Universal Protein knowledgebase in 2023. *Nucleic Acids Res.* 2023;51(D1):D523–D531. 10.1093/nar/gkac1052 36408920 PMC9825514

[ref-4] Blaxter M Mieszkowska N Di Palma F : Sequence locally, think globally: the Darwin Tree of Life Project. *Proc Natl Acad Sci U S A.* 2022;119(4): e2115642118. 10.1073/pnas.2115642118 35042805 PMC8797607

[ref-5] Buchfink B Reuter K Drost HG : Sensitive protein alignments at Tree-of-Life scale using DIAMOND. *Nat Methods.* 2021;18(4):366–368. 10.1038/s41592-021-01101-x 33828273 PMC8026399

[ref-6] Challis R Richards E Rajan J : BlobToolKit – interactive quality assessment of genome assemblies. *G3 (Bethesda).* 2020;10(4):1361–1374. 10.1534/g3.119.400908 32071071 PMC7144090

[ref-7] Cheng H Concepcion GT Feng X : Haplotype-resolved *de novo* assembly using phased assembly graphs with hifiasm. *Nat Methods.* 2021;18(2):170–175. 10.1038/s41592-020-01056-5 33526886 PMC7961889

[ref-8] Crowley L Allen H Barnes I : A sampling strategy for genome sequencing the British terrestrial Arthropod fauna [version 1; peer review: 2 approved]. *Wellcome Open Res.* 2023;8:123. 10.12688/wellcomeopenres.18925.1 37408610 PMC10318377

[ref-9] Danecek P Bonfield JK Liddle J : Twelve years of SAMtools and BCFtools. *GigaScience.* 2021;10(2): giab008. 10.1093/gigascience/giab008 33590861 PMC7931819

[ref-10] Dasch CE : Ichneumon-flies of America North of Mexico: 5. Subfamily Diplazontinae. American Entomological Institute,1964;3. Reference Source

[ref-11] Ewels P Magnusson M Lundin S : MultiQC: summarize analysis results for multiple tools and samples in a single report. *Bioinformatics.* 2016;32(19):3047–3048. 10.1093/bioinformatics/btw354 27312411 PMC5039924

[ref-12] Ewels PA Peltzer A Fillinger S : The nf-core framework for community-curated bioinformatics pipelines. *Nat Biotechnol.* 2020;38(3):276–278. 10.1038/s41587-020-0439-x 32055031

[ref-13] Fitton MG Rotheray GE : A key to the European genera of diplazontine ichneumon-flies, with notes on the British fauna. *Syst Entomol.* 1982;7(4):311–20. 10.1111/j.1365-3113.1982.tb00448.x

[ref-14] Formenti G Abueg L Brajuka A : Gfastats: conversion, evaluation and manipulation of genome sequences using assembly graphs. *Bioinformatics.* 2022;38(17):4214–4216. 10.1093/bioinformatics/btac460 35799367 PMC9438950

[ref-15] Howard C Denton A Jackson B : On the path to reference genomes for all biodiversity: lessons learned and laboratory protocols created in the Sanger Tree of Life core laboratory over the first 2000 species. *bioRxiv.* 2025. 10.1101/2025.04.11.648334 PMC12548527 41129326

[ref-16] Howe K Chow W Collins J : Significantly improving the quality of genome assemblies through curation. *GigaScience.* 2021;10(1): giaa153. 10.1093/gigascience/giaa153 33420778 PMC7794651

[ref-17] Kerpedjiev P Abdennur N Lekschas F : HiGlass: web-based visual exploration and analysis of genome interaction maps. *Genome Biol.* 2018;19(1): 125. 10.1186/s13059-018-1486-1 30143029 PMC6109259

[ref-18] Klopfstein S : Revision of the Western Palaearctic Diplazontinae (Hymenoptera, Ichneumonidae). *Zootaxa.* 2014;3801:1–143. 10.11646/zootaxa.3801.1.1 24870870

[ref-19] Klopfstein S Quicke DLJ Kropf C : The evolution of antennal courtship in diplazontine parasitoid wasps (Hymenoptera, Ichneumonidae, Diplazontinae). *BMC Evol Biol.* 2010;10(218): 218. 10.1186/1471-2148-10-218 PMC2927921 20646272

[ref-20] Klopfstein S Quicke DLJ Kropf C : Molecular and morphological phylogeny of Diplazontinae (Hymenoptera, Ichneumonidae). *Zool Scr.* 2011;40:379–402. 10.1111/j.1463-6409.2011.00481.x

[ref-21] Kurtzer GM Sochat V Bauer MW : Singularity: Scientific containers for mobility of compute. *PLoS One.* 2017;12(5):e0177459. 10.1371/journal.pone.0177459 28494014 PMC5426675

[ref-22] Lawniczak MKN Davey RP Rajan J : Specimen and sample metadata standards for biodiversity genomics: a proposal from the Darwin Tree of Life project [version 1; peer review: 2 approved with reservations]. *Wellcome Open Res.* 2022;7:187. 10.12688/wellcomeopenres.17605.1

[ref-23] Li H : Minimap2: pairwise alignment for nucleotide sequences. *Bioinformatics.* 2018;34(18):3094–3100. 10.1093/bioinformatics/bty191 29750242 PMC6137996

[ref-24] Manni M Berkeley MR Seppey M : BUSCO update: novel and streamlined workflows along with broader and deeper phylogenetic coverage for scoring of eukaryotic, prokaryotic, and viral genomes. *Mol Biol Evol.* 2021;38(10):4647–4654. 10.1093/molbev/msab199 34320186 PMC8476166

[ref-25] Merkel D : Docker: lightweight Linux containers for consistent development and deployment. *Linux J.* 2014;2014(239): 2. Reference Source

[ref-26] Morley C : The Fauna of British India, including Ceylon and Burma. Hymenoptera - Vol.III. Ichneumonidae. London: Taylor & Francis;1913.

[ref-27] O’Leary NA Cox E Holmes JB : Exploring and retrieving sequence and metadata for species across the Tree of Life with NCBI datasets. *Sci Data.* 2024;11(1): 732. 10.1038/s41597-024-03571-y 38969627 PMC11226681

[ref-28] Ranallo-Benavidez TR Jaron KS Schatz MC : GenomeScope 2.0 and Smudgeplot for reference-free profiling of polyploid genomes. *Nat Commun.* 2020;11(1): 1432. 10.1038/s41467-020-14998-3 32188846 PMC7080791

[ref-29] Rao SSP Huntley MH Durand NC : A 3D map of the human genome at kilobase resolution reveals principles of chromatin looping. *Cell.* 2014;159(7):1665–1680. 10.1016/j.cell.2014.11.021 25497547 PMC5635824

[ref-30] Rhie A McCarthy SA Fedrigo O : Towards complete and error-free genome assemblies of all vertebrate species. *Nature.* 2021;592(7856):737–746. 10.1038/s41586-021-03451-0 33911273 PMC8081667

[ref-31] Rhie A Walenz BP Koren S : Merqury: reference-free quality, completeness, and phasing assessment for genome assemblies. *Genome Biol.* 2020;21(1): 245. 10.1186/s13059-020-02134-9 32928274 PMC7488777

[ref-32] Schoch CL Ciufo S Domrachev M : NCBI taxonomy: a comprehensive update on curation, resources and tools. *Database (Oxford).* 2020;2020: baaa062. 10.1093/database/baaa062 32761142 PMC7408187

[ref-33] Scott EI : An account of the developmental stages of some aphidophagous Syrphidae (Dipt.) And their parasites (Hymenopt.). *Ann Appl Biol.* 1939;26(3):509–32. 10.1111/j.1744-7348.1939.tb06987.x

[ref-34] Twyford AD Beasley J Barnes I : A DNA barcoding framework for taxonomic verification in the Darwin Tree of Life project [version 1; peer review: 2 approved]. *Wellcome Open Res.* 2024;9:339. 10.12688/wellcomeopenres.21143.1 39386966 PMC11462125

[ref-35] Uliano-Silva M Ferreira JGRN Krasheninnikova K : MitoHiFi: a python pipeline for mitochondrial genome assembly from PacBio high fidelity reads. *BMC Bioinformatics.* 2023;24(1): 288. 10.1186/s12859-023-05385-y 37464285 PMC10354987

[ref-36] Vasimuddin M Misra S Li H : Efficient architecture-aware acceleration of BWA-MEM for multicore systems.In: *2019 IEEE International Parallel and Distributed Processing Symposium (IPDPS).*IEEE,2019;314–324. 10.1109/IPDPS.2019.00041

[ref-37] Zhou C McCarthy SA Durbin R : YaHS: Yet another Hi-C Scaffolding tool. *Bioinformatics.* 2023;39(1): btac808. 10.1093/bioinformatics/btac808 36525368 PMC9848053

