# Supplementary material for: The genome sequence of the ichneumonid wasp, Sussaba pulchella (Holmgren, 1858) (Hymenoptera: Ichneumonidae)
Source: Wellcome Open Res. 2025 Oct 10;10:564. [Version 1] doi: 10.12688/wellcomeopenres.24968.1 (PMC12627938; doi:10.12688/wellcomeopenres.24968.1)
Supplement: Peer review history

# Reviewer report: Reviewer response for version 1

**DOI:** 10.21956/wellcomeopenres.27503.r138539

**Published:** 2025-11-18

**Authors:** Taslima Sheikh

**Affiliations:** 1 Baba Ghulam Shah Badshah University, Rajouri, Jammu and Kashmir, India; 2 zoology, Baba Ghulam Shah Badshah University (Ringgold ID: 209367), Jammu, India

This data note reports a high-quality genome assembly and annotation for the ichneumonid wasp *Sussaba pulchella*. The study is well executed and makes a useful addition to genomic resources within the Hymenoptera. The methods used (PacBio HiFi sequencing, Hi-C scaffolding, and mitochondrial genome assembly) are appropriate and produce robust results.

 Only a few **minor revisions** are needed:

1. Correct the typographical error where the species name appears once as *S. puchella*.
2. Clarify that the male and female morphological characters described in the Background refer specifically to *S. pulchella*, and mention whether the combination is diagnostic.
3. Provide a short explanation for the unusually large mitochondrial genome (34.69 kb) ; for example, whether it represents true duplication, tandem repeats, or an assembly artifact  and confirm with coverage evidence if available.
4. Add the tissue type used for DNA extraction and COI barcoding, and if possible, include the approximate DNA input quantity for the library.

With these small additions, the article will be fully sound and suitable for indexing

**Recommendation:** Accept after minor revision.

Are sufficient details of methods and materials provided to allow replication by others?

Yes

Is the rationale for creating the dataset(s) clearly described?

Yes

Are the datasets clearly presented in a useable and accessible format?

Yes

Are the protocols appropriate and is the work technically sound?

Yes

Reviewer Expertise:

Entomology; Lepidoptera; Orthoptera; Hymenoptera

I confirm that I have read this submission and believe that I have an appropriate level of expertise to confirm that it is of an acceptable scientific standard.

# Reviewer report: Reviewer response for version 1

**DOI:** 10.21956/wellcomeopenres.27503.r136058

**Published:** 2025-11-05

**Authors:** Erinn Fagan-Jeffries

**Affiliations:** 1 The University of Adelaide, Adelaide, Australia

This article gives a clear introduction to the taxonomy and biology of *Sussaba pulchella*, the ichneumonid wasp for which a genome assembly is presented. The DNA sequencing and bioinformatic pipelines are included and referenced appropriately, and summary statistics of the genome assembly are presented in legible figures. 

 Specific suggestions for minor improvements to the article text, none of which are required for the article to be scientifically sound:

 1) where the article introduction states "Females can be separated from other species with the metasoma weakly laterally compressed..." I assume this is talking about *S. pulchella*, but the sentence would be less ambiguous if that was included (i.e., "Females of *S. pulchella *can be separated from other species of *Sussaba *with the metasoma..."). Similarly for the last sentence of this paragraph that starts "Males have uniformly..." it would be helpful to explicitly include if the listed characters in combination are diagnostic of the species or not.

 2) In the methods where it states "a small sample was dissected from the specimen" - it is unusual not to see the body part listed (e.g., is this a leg, part of the abdomen, etc.). I guess not necessary, but useful for future reference perhaps?

Are sufficient details of methods and materials provided to allow replication by others?

Yes

Is the rationale for creating the dataset(s) clearly described?

Yes

Are the datasets clearly presented in a useable and accessible format?

Yes

Are the protocols appropriate and is the work technically sound?

Yes

Reviewer Expertise:

Systematics of Ichneumonoidea

I confirm that I have read this submission and believe that I have an appropriate level of expertise to confirm that it is of an acceptable scientific standard.
